# Vertically-Ordered Mesoporous Silica Film Based Electrochemical Aptasensor for Highly Sensitive Detection of Alpha-Fetoprotein in Human Serum

**DOI:** 10.3390/bios13060628

**Published:** 2023-06-06

**Authors:** Tongtong Zhang, Luoxiang Yang, Fei Yan, Kai Wang

**Affiliations:** 1 Key Laboratory of Integrated Oncology and Intelligent Medicine of Zhejiang Province, Department of Hepatobiliary and Pancreatic Surgery, Affiliated Hangzhou First People’s Hospital, Zhejiang University School of Medicine, Hangzhou 310006, China; tongtongzhang@zju.edu.cn; 2 Key Laboratory of Surface & Interface Science of Polymer Materials of Zhejiang Province, Department of Chemistry, Zhejiang Sci-Tech University, Hangzhou 310018, China; 202020104156@mails.zstu.edu.cn

**Keywords:** vertically-ordered mesoporous silica films, alpha fetoprotein, electrochemistry, aptasensor

## Abstract

Convenient and rapid detection of alpha fetoprotein (AFP) is vital for early diagnosis of hepatocellular carcinoma. In this work, low-cost (0.22 USD for single sensor) and stable (during 6 days) electrochemical aptasensor was developed for highly sensitive and direct detection of AFP in human serum with the assist of vertically-ordered mesoporous silica films (VMSF). VMSF has silanol groups on the surface and regularly ordered nanopores, which could provide binding sites for further functionalization of recognition aptamer and also confer the sensor with excellent anti-biofouling capacity. The sensing mechanism relies on the target AFP-controlled diffusion of Fe(CN)_6_^3−/4−^ redox electrochemical probe through the nanochannels of VMSF. The resulting reduced electrochemical responses are related to the AFP concentration, allowing the linear determination of AFP with a wide dynamic linear range and a low limit of detection. Accuracy and potential of the developed aptasensor were also demonstrated in human serum by standard addition method.

## 1. Introduction

As one of the most common cancers in the world, hepatocellular carcinoma (HCC) will cause the liver cirrhosis and even death [1]. Compared with the imaging and histology commonly used in clinic, detection of alpha fetoprotein (AFP) concentration in serum is more simple and also has closely associated with HCC [2,3]. The content of AFP in healthy human is lower than 25 ng/mL [4], however, high levels of AFP (~500 ng/mL) can be observed in the serum of HCC patients [5]. Therefore, developing convenient and highly sensitive methods for monitoring the AFP concentration in human serum is very important for early diagnosis and prognostic evaluation of HCC.

Currently, varieties of analytical approaches have been designed to detect AFP, such as enzyme-linked immunosorption assay (ELISA) [6], electrochemistry [7], resonance light scattering [8], photoelectrochemistry [9], surface enhanced Raman scattering [10], fluorescence [11] and mass spectrometry [12]. Among them, electrochemical sensors have aroused the increasing attention due to their time-saving, low-cost, high sensitivity and easy operations [13,14,15], showing great clinical application potential in the early diagnosis of HCC [16]. To realize the specific determination, biological recognition elements including antibody, enzyme, and aptamer have been employed to be immobilized on the electrode surface [17,18,19,20,21]. Aptamer artificially screened and synthesized in vitro by systematic evolution of ligand by exponential enrichment (SELEX) displays several advantages with respect to the small size, low-cost, high stability and strong affinity towards preselected targets, which has been widely combined with functional nanomaterials to construct highly specific and sensitive electrochemical aptasensors [22]. In addition, biological macromolecules existed in human serum will cause severe bio-fouling issue on the electrodes, significantly impairing the accuracy and stability of electrodes and limiting their practical application in direct analysis of human serum. Therefore, direct and anti-fouling electrochemical determination of AFP in human serum without tedious pretreatment process still remains as a challenge.

Nanostructured mesoporous silica nanoparticles emerged as therapeutic nanocarriers have been widely utilized in the field of smart chemotheropy [23,24]. In comparison, vertically-ordered mesoporous silica films (VMSF) with perpendicularly oriented nanochannels have an apparent advantage of favored and fast mass transfer from the bulk solution to the electrode surface through nanochannels. During past decade, VMSF have shown great achievements in the construction of anti-fouling and anti-interference electrochemical sensors, due to their unique characteristics of uniform and ultrasmall pore size (usually 2~3 nm), high pore density (~40,000 μm^−2^), and mechanical stability [25,26,27,28,29]. Apart from direct analysis of small redox targets [30,31,32,33,34], researchers have also employed the recognition elements (e.g., aptamer and antibody) to develop novel VMSF-based electrochemical/electrochemiluminescent sensors for specific determination of ions [35], disease-related biomarkers [36], and cancer cell [37]. These sensors are often realized by targets-controlled diffusion of electrochemical/electrochemiluminescent probes through the nanochannels of VMSF [38,39,40,41,42]. In comparison with homogeneous sensors, immobilization of targets-specific recognition elements on the surface of VMSF spares the reagents and reduces the cost [43,44], which however remains challenge that the mass transfer through the nanochannels to the underlying electrode will be influenced by steric effect. On the basis of above issue, aptamer with small size (one tenth of antibody) becomes the prospective recognition element for developing highly specific and sensitive VMSF-based sensors.

In this work, we present a cost-effective and highly sensitive electrochemical aptasensor for specific determination of AFP by grafting AFP-specific aptamer on the VMSF surface. VMSF attached to the indium tin oxide electrode (ITO) is prepared by Stöber-solution growth method and rich of silanol groups, allowing the further functionalization of aptamer to produce the sensing interface with high specificity and affinity. When generating the complex between aptamer and AFP, the ingress of electrochemical redox (Fe(CN)_6_^3−/4−^) to the nanochannels of VMSF was hindered, which could result in the decreased electrochemical responses and realize the quantitative detection of AFP. Moreover, considering the high selectivity of aptamer and anti-fouling characteristic of VMSF, accuracy and potential of proposed electrochemical aptasensor in human serum samples are examined.

## 2. Materials and Methods

### 2.1. Materials and Reagents

All antigens including alpha-fetoprotein (AFP), carcinoembryonic antigen (CEA), carbohydrate antigen 125 (CA125) and carbohydrate antigen 199 (CA199) were all obtained from KEY-BIO Biotech Co., Ltd. (Beijing, China). S-100 antigen was purchaed from Proteintech (North America). Amino-functionalized AFP aptamer was synthesized by Sangon Biotech Co., Ltd. (Shanghai, China), with the sequence of 5′-NH_2_-GTG ACG CTC CTA ACG CTG ACT CAG GTG CAG TTC TCG ACT CGG TCT TGA TGT GGG TCC TGT CCG TCC GAA CCA ATC-3′. Sodium dihydrogen phosphate dihydrate (NaH_2_PO_4_•2H_2_O) was purchased from Mackin (Shanghai, China). Other reagents including tetraethyl orthosilicate (TEOS), hexadecyl trimethyl ammonium bromide (CTAB), (3-glycidoxypropyl)methyldiethoxysilane (GPTMS), bovine albumin (BSA) sodium phosphate dibasic dodecahydrate (Na_2_HPO_4_•12H_2_O) were all bought from Aladdin (Shanghai, China). And phosphate buffer (PBS) was prepared with NaH_2_PO_4_•2H_2_O and Na_2_HPO_4_•12H_2_O. All reagents used were analytical reagent without further treatment. Human blood serum was provided by Affiliated Hangzhou First People’s Hospital (Hangzhou, China). The ultrapure water (18.2 MΩ cm) was prepared from Mill-Q system.

### 2.2. Experiments and Instrumentations

The thickness and the pore structure of VMSF were characterized by scanning electron microscope (SEM, SU8010, Hitachi, Tokyo, Japan) and transmission electron microscope (TEM, HT7700, Hitachi, Japan), respectively. The accelerating voltage used for SEM and TEM measurements were 3 kV and 100 kV. Electrochemical characterization contains cyclic voltammetry (CV), differential pulse voltammetry (DPV), and electrochemical impedance spectroscopy (EIS), which was conducted on the electrochemical workstation (PGSTAT302N, Metrohm, Herisau, Switzerland). The scan rate of CV was 50 mV/s, and the step and modulation amplitude of DPV were 0.005 V and 0.025 V respectively. The error bars in the measurements were calculated from the standard deviation of three experiments.

### 2.3. Modification of VMSF on ITO Electrode

Prior to the growth of VMSF, bare ITO electrodes need to be pretreated by placing bare ITO electrodes into 1 M NaOH solution overnight, and then into acetone, ethanol and water under ultrasonication for 30 min, respectively. After rinsed by amounts of ultrapure water and dried by N_2_ atmosphere, the freshly cleaned bare ITO electrodes were obtained and used to accomplish the growth of VMSF using Stöber-solution growth approach [45,46]. The detailed procedure was as follows: CTAB (160 mg) was dissolved in 100 mL 70% ethanol aqueous solution and ammonia (2.5%, 100 μL) and TEOS (80 μL) were added in turn to achieve silica-based precursor solution. Bare ITO electrodes (2.5 cm × 5 cm) were placed into the above precursor solution with sealing treatment and incubated in a water bath at 60 °C for 24 h. After the reaction finished, the resulting electrode confined surfactant micelles (SM) into the nanochannels, termed as SM@VMSF/ITO electrodes, were washed with ultrapure water and further aged at 100 °C for 10 h. SM could be extracted from silica nanochannels by using 0.1 M HCl-ethanol solution to obtain VMSF/ITO electrodes.

### 2.4. Construction of Electrochemical Aptasensor

**Figure 1:**
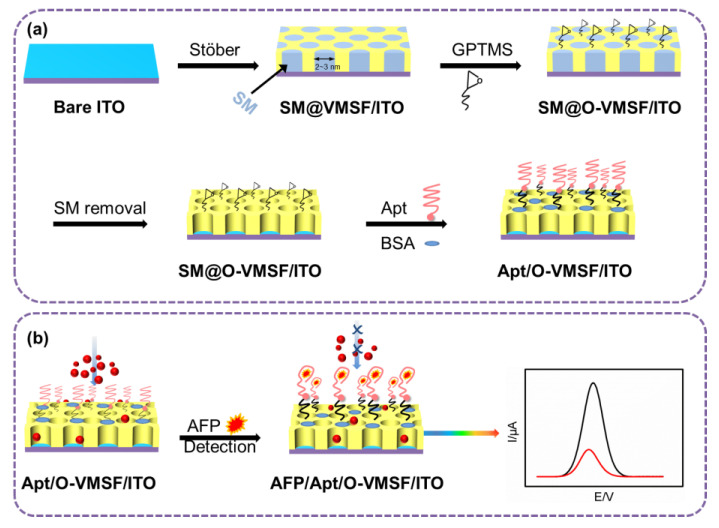
Schematic illustration for the fabrication of electrochemical aptasensing interface (**a**) and AFP detection (**b**).

VMSF/ITO based electrochemical aptasensor for AFP detection was constructed as the procedure shown in Figure 1a, which involved in the surface modification of GPTMS and AFP-specific aptamer. Firstly, the SM@VMSF/ITO electrode was placed into ethanol solution containing 0.052% (*v*/*v*) GPTMS for 1 h, allowing the modification of GPTMS on the outer surface of SM@VMSF/ITO based on the silanization reaction and producing epoxy groups for further functionalization. Subsequently, in order to remove the micelles from the nanochannels of VMSF, the resulting electrode was stirred in HCl-ethanol (0.1 M) solution for 5 min to obtain GPTMS modified VMSF/ITO, named as O-VMSF/ITO electrode. Secondly, the AFP aptamer (50 μL, 0.1 μM) was dropped onto the surface of O-VMSF/ITO at 4 °C for 2.5 h. After the residual aptamer was washed with PBS (0.01 M, pH = 7.4), BSA (0.5%) was used to block the nonspecific active sites at 4 °C for 0.5 h. Finally, the constructed electrochemical aptasensing interface was called Apt/O-VMSF/ITO.

### 2.5. Electrochemical Determination of AFP

K_3_[Fe(CN)_6_]/K_4_[Fe(CN)_6_] (2.5 mM) in KCl (0.1 M) solution was used as the electrochemical probe for the electrochemical determination of AFP. Different concentration of AFP (50 μL) was dropped onto the electrochemical aptasensing interface (Apt/O-VMSF/ITO) and incubated at 4 °C for 1 h. After rinsing with PBS (0.01 M, pH = 7.4) to remove the residual AFP, AFP was specifically recognized and bounded to the Apt/O-VMSF/ITO electrode surface, resulting in the decreased electrochemical signals of Fe(CN)_6_^3−/4−^. The variation of electrochemical signals of Fe(CN)_6_^3−/4−^ was recorded using DPV method, realizing the determination of AFP. To further evaluate the reliability of constructed electrochemical aptasensor in clinical detection, standard addition method was chosen to detect AFP in human serum samples.

## 3. Results and Discussion

### 3.1. Construction of Electrochemical Aptasensor

Figure 1a shows the construction process of the electrochemical aptasensor, which involves in the surface modification of GPTMS and AFP-specific aptamer. As seen, SM@VMSF/ITO electrode directly prepared by Stöber-solution growth method is first modified with GPTMS, resulting in the epoxy groups onto the outer surface of VMSF (SM@O-VMSF/ITO). Then, SM is excluded from the nanochannels of VMSF to obtain O-VMSF/ITO electrode with open channels. AFP-specific aptamer is immobilized on the O-VMSF/ITO electrode surface through silanization reaction between amino groups of aptamer and epoxy groups of GPTMS. After blocking the nonspecific active sites by blocking agent (BSA), electrochemical aptasensing interface is prepared, designated as BSA/Apt/O-VMSF/ITO. As shown in Figure 1b, the access of Fe(CN)_6_^3−/4−^ to the underlying ITO electrode through silica nanochannels is observed. And formed AFP-aptamer complex could inhibit the entrance of Fe(CN)_6_^3−/4−^ into the silica nanochannels, leading to the reduced electrochemical responses and ultimately allowing the determination of AFP.

### 3.2. Morphological and Electrochemical Characterizations of VMSF

**Figure 2:**
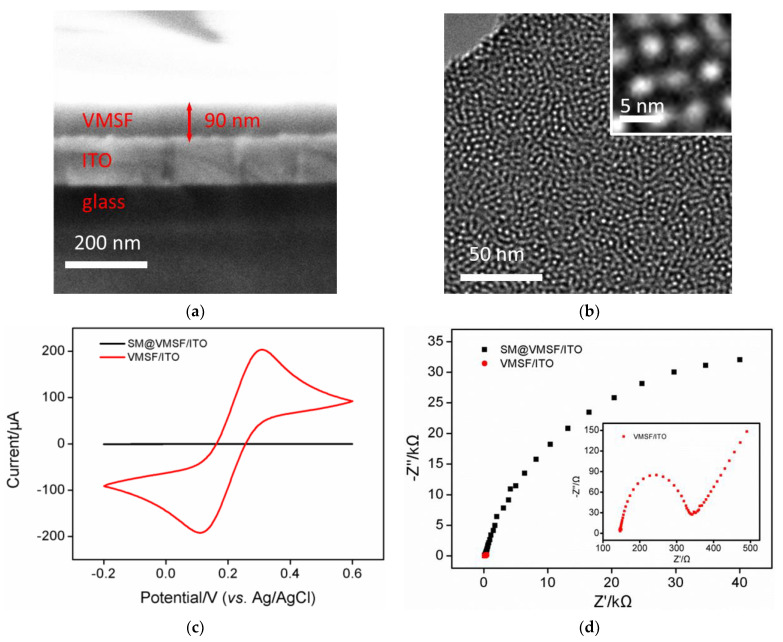
Cross-sectional SEM (**a**) and top-view TEM (**b**) images of VMSF. (**c**) CV curves obtained at SM@VMSF/ITO and VMSF/ITO electrodes in 0.1 M KCl solution containing 2.5 mM K_3_Fe(CN)_6_/K_4_Fe(CN)_6_. (**d**) Nyquist plots of SM@VMSF/ITO and VMSF/ITO electrodes obtained in 0.1 M KCl solution containing 2.5 mM K_3_Fe(CN)_6_/K_4_Fe(CN)_6_.

SEM and TEM were used to characterize the morphology of VMSF, as shown in Figure 2a,b. The cross-sectional SEM image of VMSF/ITO electrode shows that VMSF with homogeneous thickness (~90 nm) is on the top of ITO coated glass (Figure 2a). The top-view TEM exhibits that regularly oriented and uniform silica nanopores are observed as bright dots and their diameter measured by Image J software are about 2~3 nm (Figure 2b), which is smaller than that of various biological fouling agents in human serum and could be acted as efficient anti-fouling layer. It can be found from the cross-sectional view TEM image that vertical silica nanochannels is parallel to each other and the lengh of silica nanochannels is 90 nm (Figure S1), which is in a accordance to the result from Figure 2a. CV and EIS were then used to examine the integrity of VMSF on the ITO electrode. As shown in Figure 2c, there is almost no redox peak current of the Fe(CN)_6_^3−/4−^ probe before SM removal, which is due to the hydrophobic SM filled in the channel inhibits the mass transfer of the hydrophilic and charged Fe(CN)_6_^3−/4−^. After SM removal, VMSF possesses open nanochannels and Fe(CN)_6_^3−/4−^ probe could access to the underlying ITO electrode through the nanochannels, producing a pair of redox peaks. The semicircle in the high-frequency region refers to the electron transfer-limited process and its equivalent diameter reflects the apparent charge-transfer resistance (*R*_ct_). As presented in Figure 2d, *R*_ct_ of VMSF/ITO electrode is much smaller than that of SM@VMSF/ITO electrode, indicating the integrity and permeability of the VMSF modified on the electrode.

### 3.3. Electrochemical Characterization of the Construction Process of Electrochemical Aptasensor

**Figure 3:**
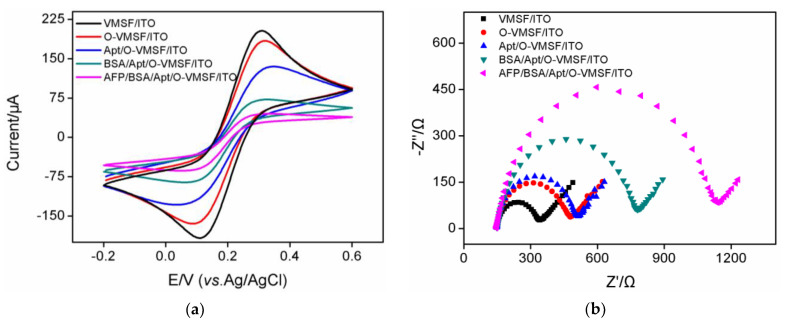
CV curves (**a**) and EIS plots (**b**) of VMSF/ITO, O-VMSF/ITO, Apt/O-VMSF/ITO, BSA/Apt/O-VMSF/ITO and AFP/BSA/Apt/O-VMSF/ITO electrodes obtained in 0.1 M KCl containing 2.5 mM K_3_Fe(CN)_6_/K_4_Fe(CN)_6_. The concentration of AFP is 1 ng/mL.

Electrochemical probe, Fe(CN)_6_^3−/4−^, was employed to investigate the construction process of our proposed electrochemical aptasensor using CV and EIS methods and the results were shown in Figure 3. As seen in Figure 3a, modification of VMSF with GPTMS, aptamer and further BSA leads to the progressively decreased redox responses, which is ascribed to the steric hindrance impedes the diffusion of Fe(CN)_6_^3−/4−^ to the underlying ITO electrode through silica nanochannels. And the redox responses of Fe(CN)_6_^3−/4−^ further decrease after incubation with AFP, indicating the successful preparation of electrochemical aptasensing interface. Similar phenomenon could be observed from EIS data displayed in Figure 3b, modification of VMSF with GPTMS, aptamer, BSA and further binding of AFP results in a progressive increase of *R*_ct_, suggesting the feasibility of our developed electrochemical aptasensor for AFP determination.

### 3.4. Optimized Conditions for the Construction of Electrochemical Aptasensor

**Figure 4:**
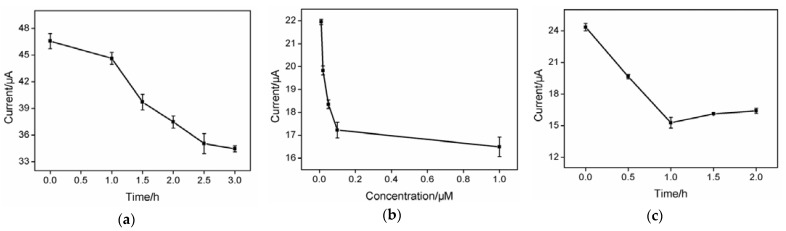
Effect of coupling time for aptamer immobilization (**a**), aptamer concentration (**b**) and incubation time for AFP (**c**) on current signal value.

In order to improve the sensitivity of AFP detection, effect of coupling time of aptamer onto the surface of the O-VMSF/ITO electrode was first studied. As shown in Figure 4a, in the range of 0–3 h, the anodic peak current of Fe(CN)_6_^3−/4−^ decreases obviously with increasing the coupling time and reaches equilibrium at 2.5 h, proving the outer surface of VMSF has been successfully modified with AFP-specific aptamer. Therefore, 2.5 h is selected as the optimal coupling time for aptamer modification.

As AFP-aptamer complex was formed in the recognition process by the developed BSA/Apt/O-VMSF/ITO sensor, aptamer concentration and the incubation time between aptasensor and AFP were investigated. AFP-specific aptamers with five different concentrations were used to prepare BSA/Apt/O-VMSF/ITO aptasensor and their responses on the AFP (1.0 ng/mL) were explored. When the aptamer concentration exceeded 0.1 μM, the anodic peak current remained almost unchanged, indicating that the aptamer was fully immobilized on the sensor surface. Therefore, the aptamer concentration of 0.1 μM is selected for subsequent work (Figure 4b). As depicted in Figure 4c, the anodic peak current decreases with an increase of incubation time and reaches stable at 1.0 h. Thus, 1 h is set as the optimal interaction time between aptamer and AFP.

### 3.5. Determination of AFP

**Figure 5:**
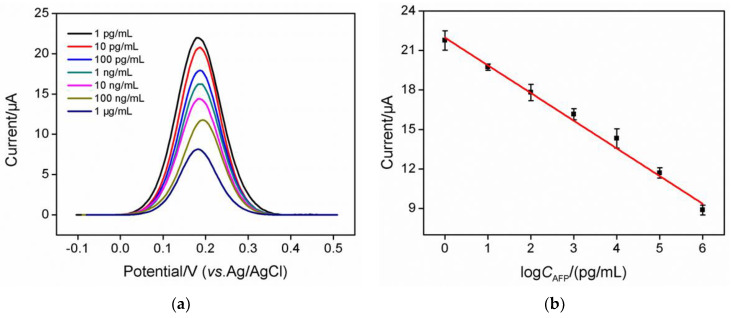
(**a**) DPV curves of the developed electrochemical aptasensor in the presence of different concentrations of AFP (1 pg/mL–1 μg/mL). (**b**) Corresponding calibration curve.

Under the optimal detection conditions, different concentrations of AFP (1 pg/mL to 1 μg/mL) were incubated with the developed BSA/Apt/O-VMSF/ITO sensor and the electrochemical responses of Fe(CN)_6_^3−/4−^ were recorded by DPV. As shown in Figure 5a, anodic peak current decreases as the AFP concentration increases, which arises from the steric hindrances formed by AFP-aptamer complex on the electrode surface. And the calibration curve for AFP detection shown in Figure 5b displays a good linear range between the anodic peak current and the logarithm of AFP concentration (log*C*_AFP_), with a regression equation of *I* (μA) = −2.10 log*C*_AFP_ (pg/mL) + 21.9 (*R*^2^ = 0.992) and a limit of detection (LOD) of 0.31 pg/mL (signal to noise ratio is 3). Such a low LOD arises from the fast mass transport inside the vertical silica nanochannels and high affinity between aptamer and AFP. Table 1 presents a comparison of our developed BSA/Apt/O-VMSF/ITO sensor with other electroanalytical techniques for AFP determination. By contrast, our proposed electrochemical aptasensor possesses a wider dynamic linear range and a lower LOD.

### 3.6. Selectivity, Reproducibility and Stability of the Developed Electrochemical Aptasensor

**Figure 6:**
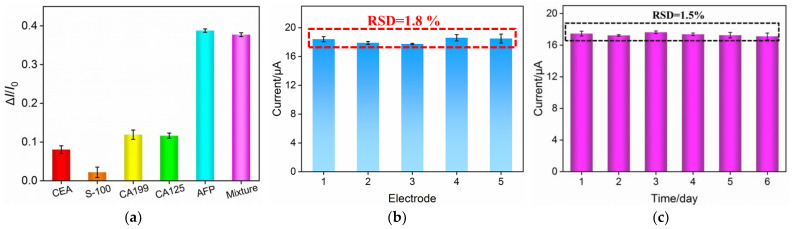
(**a**) Electrochemical responses of BSA/Apt/O-VMSF/ITO sensor to CEA, S-100, CA199, CA125, AFP or their mixture. *I*_0_ and *I* denote the anodic peak current before (*I*_0_) and after (*I*) incubation with AFP or other antigens and Δ*I* = *I* − *I*_0_. The concentration of AFP and other proteins are 0.1 ng/mL and 1 ng/mL, respectively. (**b**) Anodic peak currents obtained at five different electrodes. (**c**) Stability of the developed electrochemical aptasensor after storage for different days. The concentration of AFP in (**b**,**c**) is 1.0 ng/mL.

Selectivity, reproducibility and stability play important roles in evaluation of the developed electrochemical aptasensor. Some common antigens including CEA, CA199, S-100, CA125 and their mixture were incubated with the BSA/Apt/O-VMSF/ITO sensor and their results were shown in Figure 6a. As revealed, except for AFP, no significant responses to other proteins are observed at the proposed aptasensor, implying the satisfactory specificity of the constructed electrochemical aptasensor. To evaluate the reproducibility of the developed BSA/Apt/O-VMSF/ITO sensor, five different modified electrodes were prepared and used to detect 1.0 ng/mL AFP. As presented in Figure 6b, the relative standard deviation (RSD) of anodic peak current calculated from these modified electrodes was 1.8%, indicating the great reproducibility of the electrochemical aptasensor. Figure 6c studies the stability of the developed electrochemical aptasensor within 6 days. It could be found that after six-day storage, our BSA/Apt/O-VMSF/ITO sensor remains almost unchanged responses to 1.0 ng/mL AFP with an RSD of 1.5%, showing the attractive stability of the as-prepared electrochemical aptasensor.

### 3.7. Real Sample Analysis

To access the practical utility of our proposed electrochemical aptasensor, the healthy human serum was spiked with a series of known concentrations of AFP standard solution and tested by our BSA/Apt/O-VMSF/ITO sensor. As shown in Table 2, the initial concentration of AFP in healthy human serum is measured as 1.0 ng/mL, which is close to the result measured by ELISA method (1.7 ng/mL). Recoveries ranging from 98.8% to 101% and RSD values ranging from 1.7% to 2.9% are obtained, verifying that the developed electrochemical aptasensor can be served as a reliable tool for AFP detection in clinical diagnosis of HCC.

## 4. Conclusions

In summary, we reported the low-cost and simple electrochemical aptasensor for highly sensitive and selective detection of AFP in human serum based on an excellent anti-fouling layer of VMSF and a specific recognition layer of AFP-specific aptamer. Owing to the silanol groups of VMSF, AFP-specific aptamer with small size can not only covalently graft to the surface of VMSF and endow the sensing interface with selective recognition ability, but also remain the effective permeability of VMSF. With the help of electrochemical probe (Fe(CN)_6_^3−/4−^), electrochemical response variations have closely relation with AFP concentration, enabling the linear determination of AFP with a wide dynamic linear range and a low limit of detection. Furthermore, the inherent anti-fouling property of VMSF confers the proposed electrochemical aptasensor with promising feasibility in direct and selective analysis of AFP in human serum, which is potential to design a miniaturized and portable sensor integrated with smart phone. And the proposed strategy provides a convenient method for early diagnosis and prognostic evaluation of hepatocellular carcinoma.

## Figures and Tables

**Table 1:** Comparison with the existing electroanalytical techniques for the detection of AFP.

Electrode	Method	Liner Rang (ng/mL)	LOD (pg/mL)	Incubation Time (h)	Ref.
AFP/PBNP ^1^-Apt ^2^/GO/AuE	DPV	0.01–300	6.3	1.5	[47]
Apt/PtNPs/RGO ^3^-CS ^4^-Fc ^5^/AuNPs/SPCE ^6^	DPV	1–10^4^	3.013 × 10^4^	0.5	[48]
Apt/PtNPs/ GO-COOH ^7^/SPGE ^8^	SWV ^15^	3–30	1.22 × 10^3^	0.75	[49]
AFP/Apt/Peptide/ PANI ^9^/GCE	DPV	10^−6^–1	5.9 × 10^−4^	1.0	[50]
ConA ^10^-AgNPs/AFP/miR16/ESP ^11^/AuE ^12^	DPV	0.05–10	8.76	2.0	[51]
Ab_1_^13^/2D ^14^ MoSe_2_/2D WSe_2_/SPCE	DPV	0.001–5	0.75	0.75	[52]
AFP/Apt/O-VMSF/ITO	DPV	0.001–1000	0.31	1.0	This work

^1^ prussian blue nanoparticles; ^2^ aptamer; ^3^ reduced graphene oxide; ^4^ chitosan; ^5^ ferrocene; ^6^ screen-printed carbon electrode; ^7^ carboxylated-graphene oxide; ^8^ screen-printed graphene-carbon paste electrode; ^9^ polyaniline; ^10^ concanavalin A; ^11^ electrochemical sensing probe; ^12^ gold electrode; ^13^ antibody; ^14^ two dimensional; ^15^ square wave voltammetry.

**Table 2:** Determination of AFP in diluted human serum samples.

Sample	Spiked (ng/mL)	Found (ng/mL)	RSD (%) (n = 3)	Recovery (%)
Human serum *	0.100	0.100	2.9	100
	1.00	1.01	1.7	101
	10.0	9.88	1.8	98.8

* Human serum was diluted by 100-fold using 0.01 M PBS (pH 7.4) solution.

## Data Availability

The data presented in this study are available on request from the corresponding author.

## Supplementary Materials

The following supporting information can be downloaded at: https://www.mdpi.com/article/10.3390/bios13060628/s1, Figure S1: TEM characterization of VMSF.
