# Supplementary material for: Vertically-Ordered Mesoporous Silica Film Based Electrochemical Aptasensor for Highly Sensitive Detection of Alpha-Fetoprotein in Human Serum
Source: Biosensors (Basel). 2023 Jun 6;13(6):628. doi: 10.3390/bios13060628 (PMC10296332; doi:10.3390/bios13060628)
Supplement: Supplementary file 1 [file biosensors-13-00628-s001.zip › biosensors-2342306-supplementary.pdf]

biosensors

Supporting Information to

Vertically-ordered mesoporous silica film based electrochemical aptasensor for highly sensitive detection of alpha-fetoprotein in human serum

Tongtong Zhang [1,†], Luoxiang Yang [2,†], Fei Yan [2,*] and Kai Wang [1,*]

[1] Key Laboratory of Integrated Oncology and Intelligent Medicine of Zhejiang Province, Department of Hepatobiliary and Pancreatic Surgery, Affiliated Hangzhou First People's Hospital, Zhejiang University School of Medicine, Hangzhou 310006, China; tongtongzhang@zju.edu.cn
[2] Key Laboratory of Surface & Interface Science of Polymer Materials of Zhejiang Province, Department of Chemistry, Zhejiang Sci-Tech University, Hangzhou 310018, China; 202020104156@mails.zstu.edu.cn
* Correspondence: yanfei@zstu.edu.cn (F.Y.); kaiw3@zju.edu.cn (K.W.)
† These authors contributed equally to this work.

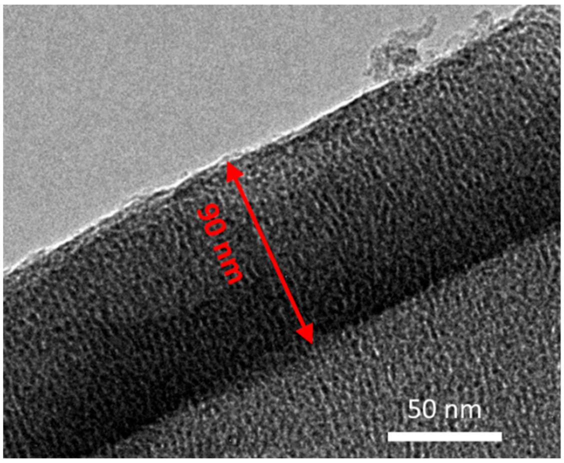

Figure S1. Cross-sectional TEM image of VMSF.
